# Association between age, gender, body mass index, and pulmonary function in preoperative patients with lung cancer

**DOI:** 10.1111/crj.13476

**Published:** 2022-01-26

**Authors:** Weicheng Xu, Yakang Liu, Bin Zeng, Xinping Li

**Affiliations:** ^1^ Department of Physical Medicine and Rehabilitation, Guangdong Geriatric Institute Guangdong Academy of Medical Sciences and Guangdong Provincial People's Hospital Guangzhou China

**Keywords:** association, confounding factor, pulmonary function, small airway, structural equation model

## Abstract

**Introduction:**

Many confounding factors such as sex, age, and body mass index (BMI) affect pulmonary function parameters, but there are limited data about the direct and/or indirect effects of small airway function on lung function for differences in confounding factors.

**Objectives:**

This study aimed to use structural equation model (SEM) to explain the influence of the confounding factors (age, sex, and BMI) on the relationship between small airway function and lung function in patients with lung cancer.

**Methods:**

A cross‐sectional observational study was conducted in a single medical center. Subjects were assessed; small airway function was specified by MEF25% and MEF50%; lung function by FVC; pulmonary obstruction by FEV1, FEV1%, and FEV1/FVC; and PEF and PEF% reflected the strength of abdominal muscles. The measurement model was analyzed by confirmatory factor analysis. The SEM was conducted to analyze the structural models of the effects of the confounding factors.

**Results:**

In the measurement model, variables were fit to their domains, the path linking age and sex to pulmonary obstruction was positive and statistically significant, and the path linking sex to muscle strength was also positive and statistically significant. Muscle strength positively and significantly mediates the path between sex and FVC. As a moderator, BMI increased the effects of small airway function on FVC.

**Conclusion:**

Age and sex were directed to pulmonary obstruction, and muscle strength as a mediator between sex and lung function was novel, and BMI adjusted the effects of small airway function on FVC.

## 1 INTRODUCTION

Surgical resection is an important therapy for many lung cancer patients. In order to decrease the risk of mortality and postoperative dyspnea, the invasive treatment requires a careful preoperative assessment protocol including pulmonary function.^1^ However, many confounding factors such as sex, age, and body mass index (BMI) affect pulmonary function parameters,^2^ making it difficult to inform important treatment decisions. In addition, the influence paths of those factors on pulmonary function are still a matter of debate.

The lung peripheral specialized structure is major determinants of overall lung function. Small airway function is associated with asthma symptoms, and the normal changes in lung volume are thought to be partly due to changes in peripheral airway structure. To examine the factors on small airway function may provide novel views to change in pulmonary function. And increasing evidence shows that sex, age, and body mass status affect small airway function.^3^, ^4^, ^5^, ^6^, ^7^, ^8^, ^9^, ^10^ Among the subjects without airflow obstruction, aging is associated with functional small airway abnormality.^3^ Changes in FEV1/FVC in healthy adults are associated with the changes in peripheral airway function with aging.^4^ Sandra Ekström et al.^5^ found that BMI status in childhood and adolescence was associated with evidence of airway obstruction including the small airways. Furthermore, genetic effects contributed significantly to the subject differences observed in FEV1/FVC and PEF.^6^ And sex‐related differences in bronchial parameters are also present in chronic obstructive pulmonary disease (COPD).^7^ However, only a few studies explored the effects of those factors (age, sex, and BMI) on the relationships between small airway function and lung function in patients with lung cancer.

Considering the variables available, testing the direct and/or indirect effects of small airway function on lung function for differences in confounding factors (sex, age, and BMI) was just a theoretical model. To test the complex multifactorial theoretical model and establish the models for describing the relationships between the variables available, the structural equation model (SEM) could be used.^11^ Therefore, the objective of this study to examine the associations between small airway function and lung function, and their relationship with age, sex, and BMI status in preoperative patients with lung cancer. In this study, small airway function was specified by MEF25% and MEF50%; lung function by FVC; and pulmonary obstruction by FEV1, FEV1%, and FEV1/FVC. And PEF and PEF% reflected the strength of abdominal muscles^12^; see Table 1 for further information. The present study may contribute to understanding the contribution of small airway function to changes in pulmonary function with aging, sex, and BMI status.

**TABLE 1:** Internal consistency reliability and convergent validity

Latent variables	Indicators	Indicator description	Loading	CR	AVE
Small airway function	MEF25	Maximal expiratory flow 25%	0.91	0.92	0.86
	MEF50	Maximal expiratory flow 50%	0.94		
Lung function	FVC	Forced vital capacity	1.00	1.00	1.00
Muscle strength	PEF	Forced expiratory flow	0.92	0.87	0.77
	PEFp	Predicted PEF	0.84		
Pulmonary obstruction	FEV1	Forced expiratory volume in first second	0.80	0.85	0.65
	FEV1p	Predicted FEV1	0.72		
	FEV1/PVCp	FEV1/FVC ratio	0.89		

Abbreviations: AVE, average variance extracted; CR, composite reliability.

## 2 METHODS

### 2.1 Procedure

Ethical approval has been obtained from the Guangdong Provincial People's Hospital. The present study was conducted according to the Declaration of Helsinki ethical principles for human experimentation. A cross‐sectional observational study was designed. The stage T1,T2, or T3a lung cancer patients with or without smoking were enrolled in this study. Patients were excluded if they were diagnosed with the stage T3b and T4 lung cancer, associated with other serious chronic disease, or refused to participate in the study. A total of 100 subjects who had undergone pulmonary function tests in our hospital between January 2020 and December 2020 were enrolled in this study.

### 2.2 Pulmonary function measurements

Pulmonary volumes were measured using a spirometer (Electgraph HI‐101, CHEST, Tokyo, Japan), and corrected for temperature and barometric pressure, according to the American Thoracic Society recommendations. Each patient performed at least three trials and the best performance was used for analysis.

### 2.3 Statistical analysis

Data were expressed as the mean values ± SD for ordinal or continuous variables, and as numbers and percentages for categorical variables. Statistical analysis was performed using GraphPad Prism software Version 8.0 for Windows (GraphPad Software, San Diego, California, USA). Then we used SmartPLS Version 3.2 to analyze the data for more robust structural equation analyses even from fewer data distributions. The path analysis method was conducted in a two‐step analysis approach: Step 1, selecting observed variables into measurement model and examining the measurement model; Step 2, to examine the path coefficient (*β*), the effect sizes (*f* ^2^), and the determination of coefficient (*R* ^2^) of each independent variable of the dependent variables. Finally, a mediator or moderator analysis was executed using a bootstrapping procedure. There is not a consensus on sample size for SmartPLS, which is robust even with a smaller sample size because it applies a bootstrapping method (5000 resamples) for determining the significance level of the weights, loadings, and path coefficient. According to the recommendation,^13^, ^14^ the sample of 100 patients with lung cancer in this study was plenty to do PLS.

## 3 RESULTS

There were a total of 100 patients including peripheral lung cancer, and all of the patients did not perform a surgery treatment. Table 2 summarizes the baseline clinical characteristics of the patients. The mean age was 59.13 ± 14.58 years. The tumor–node–metastasis (TNM) classification of the lung cancers were as follows: 62 stages 1, 30 stages 2, and 8 stages 3a. The preoperative FEV1 values were 2.22 ± 0.59 L; the preoperative FEV1/FVC values were 82.13 ± 14.21%.

**TABLE 2:** The patients' clinical characteristics and functional variables in patients (*n* = 100)

Variables	Gender/mean ± SD	Variables	Mean ± SD
Sex (male/female)	56/44	FVC	2.97 ± 0.74
Age, years	59.13 ± 14.58	FEV1	2.22 ± 0.59
BMI	22.36 ± 3.497	FEV1/FVC	82.13 ± 14.21
Smoking history (no/yes)	78/22	PEF	6.17 ± 1.68
Histological type (Ad/Sc/other)	55/33/12	PEF%	87.82 ± 27.70
Pathological stage (I/II/III)	62/30/8	MEF25%	99.58 ± 46.40
Height (cm)	164.4 ± 7.32	MEF50%	71.64 ± 32.98

Abbreviation: BMI, body mass index.

### 3.1 Evaluation of the measurement models

We utilized the factor loadings of the indicators, composite reliability (CR), and average variance extracted (AVE) to test the convergence of the observed variables. We found that all of the items were above the cut‐off of 0.62 loading, CR ranged from 0.85 to 1.00, and exceeding the recommended threshold value of 0.65 with AVE. Following the recommendation,^15^ those results indicated that the constructs were good and allowed us to proceed to test the research hypothesis, as shown in Table 1.

### 3.2 Evaluation of the structural models

**FIGURE 1:**
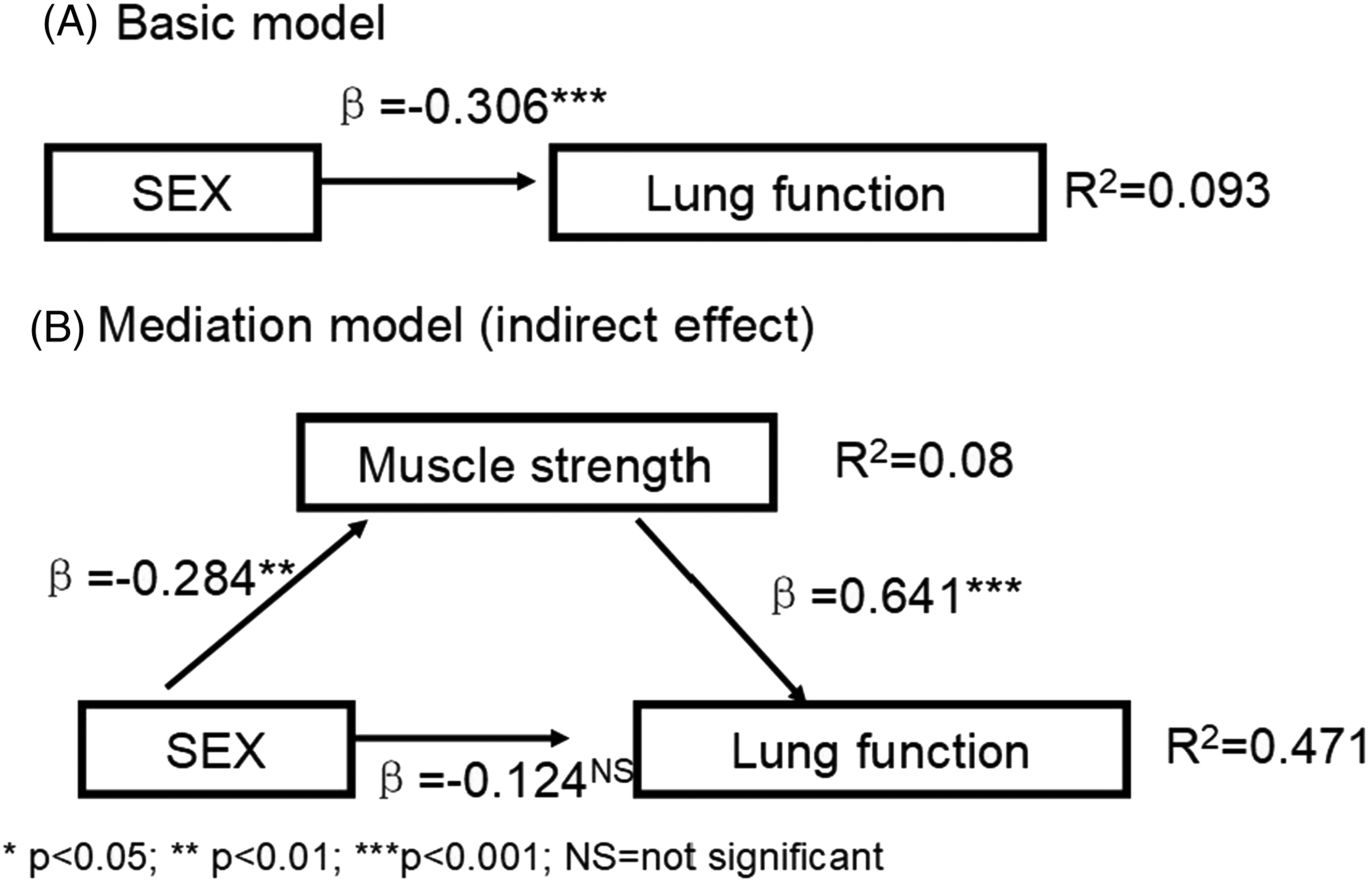
The structural model of sex on the relationships between pulmonary function in patients with lung cancer

**FIGURE 2:**
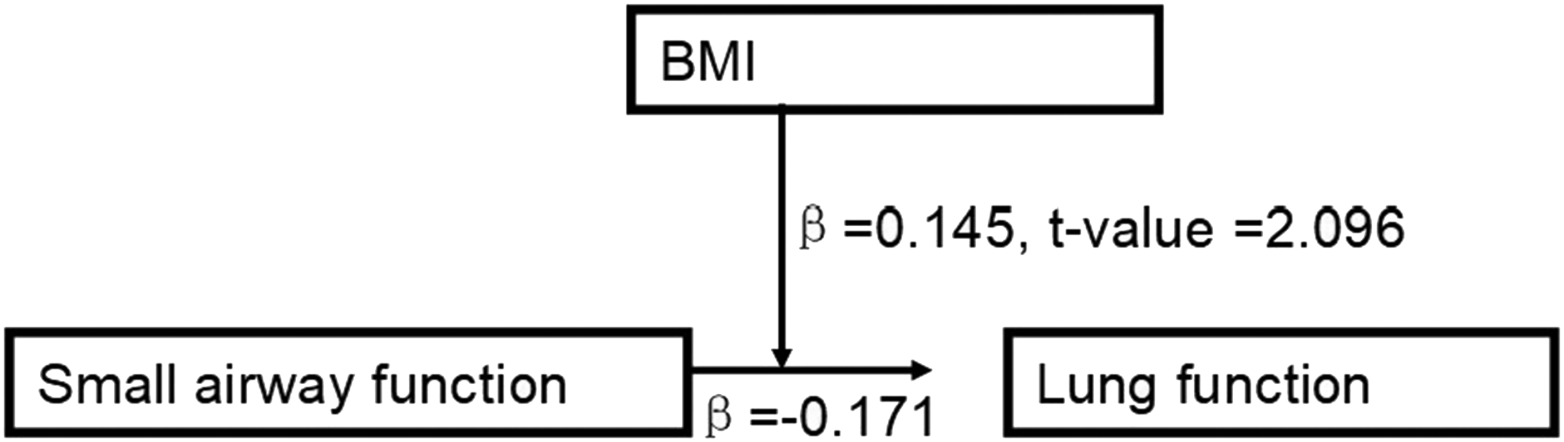
The structural model of age on the relationships between pulmonary function in patients with lung cancer. BMI, body mass index

**FIGURE 3:**
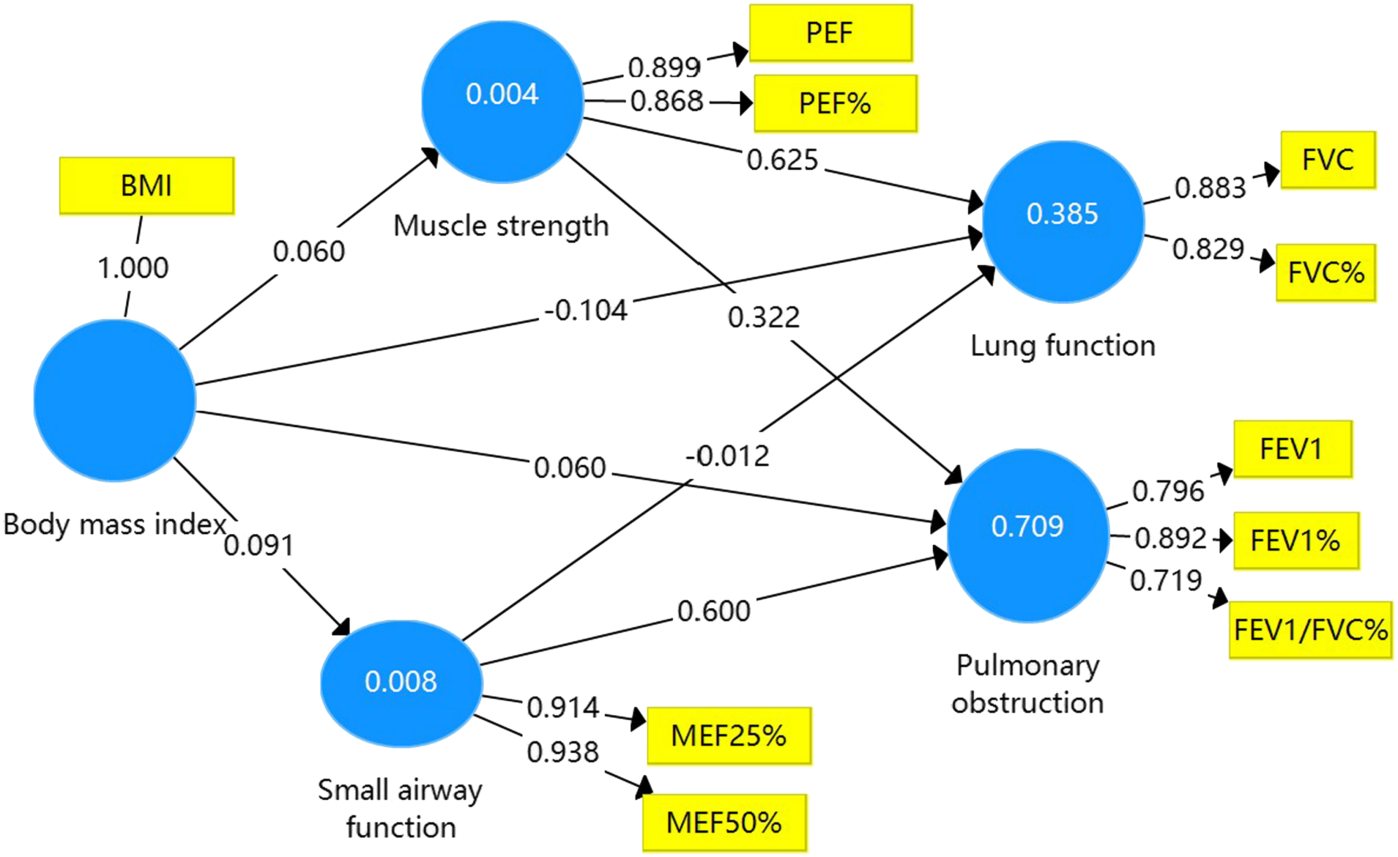
The structural model of body mass index (BMI) on the relationships between pulmonary function in patients with lung cancer

Figures 1, 2, 3 and Table 3 show the results of the path coefficient (*β*) and the coefficient of determination (*R* ^2^ value). The *R* ^2^ value for FVC is about 0.40, which means 40% of the changes in lung function were due to small airway function, muscle strength, and age, sex, or BMI in the models. This *R* ^2^ value of 0.70 for pulmonary obstruction was due to small airway function, muscle strength, and age, sex, or BMI in the model. According to the suggestions from Cohen et al,^16^ the effect of a predictor latent variable is small at the structural level if *f* ^2^ is 0.02, medium if *f* ^2^ is 0.15, and large if *f* ^2^ is 0.35. In the present study, age, sex, and BMI have a small effect on the perceived lung function and pulmonary obstruction.

**TABLE 3:** Results of effect size *f* ^2^ and *R* ^2^ analysis

Dependent construct	Independent construct	*R* ^2^	*f* ^2^	Inference
Model 1				
Lung function	Small airway function	0.379	0.051	Small
	Muscle strength		0.565	Large
	Age		0.023	Small
Pulmonary obstruction	Small airway function	0.714	0.819	large
	Muscle strength		0.259	Medium
	Age		0.032	Small
Model 2				
Lung function	Small airway function	0.384	0.002	Small
	Muscle strength		0.393	large
	Sex		0.009	Small
Pulmonary obstruction	Small airway function	0.727	0.613	Large
	Muscle strength		0.305	Medium
	Sex		0.082	Small
Model 3				
Lung function	Small airway function	0.385	0.031	Small
	Muscle strength		0.506	Large
	BMI		0.005	Small
Pulmonary obstruction	Small airway function	0.709	0.838	Large
	Muscle strength		0.238	Medium
	BMI		0.012	Small

Abbreviation: BMI, body mass index.

Table 4 shows a summary of the path results and the corresponding *t*‐values and estimated *p*‐value associated with each *t*‐value. The significance of the path coefficients (*β*) was tested by checking the significance of the *t*‐value using the bootstrap function with 5000 resamples. For all of the paths, a two‐tailed *t*‐test was used. The paths linking age, sex, and BMI to FVC were negative and statistically insignificant. The paths linking age and sex to pulmonary obstruction were positive and statistically significant, and the path linking sex to muscle strength was also positive and statistically significant.

**TABLE 4:** Summary of structural model hypotheses

Paths		Path coefficients (*β*)	*t*‐value	Sig.	Inference
Age	→ Lung function	−0.119	1.828	0.068	Not supported
	→ Muscle strength	0.000	0.002	0.999	Not supported
	→ Pulmonary obstruction	−0.096	2.127	0.034	Supported
	→ Small airway function	−0.088	1.130	0.258	Not supported
Sex	→ Lung function	−0.137	1.400	0.161	Not supported
	→ Muscle strength	−0.221	2.136	0.033	Supported
	→ Pulmonary obstruction	0.170	2.460	0.014	Supported
	→ Small airway function	0.160	1.629	0.103	Not supported
BMI	→ Lung function	−0.056	0.795	0.426	Not supported
	→ Muscle strength	0.061	0.653	0.514	Not supported
	→ Pulmonary obstruction	0.060	1.264	0.206	Not supported
	→ Small airway function	0.091	0.996	0.319	Not supported

Abbreviation: BMI, body mass index.

### 3.3 Result for mediation test

**FIGURE 4:**
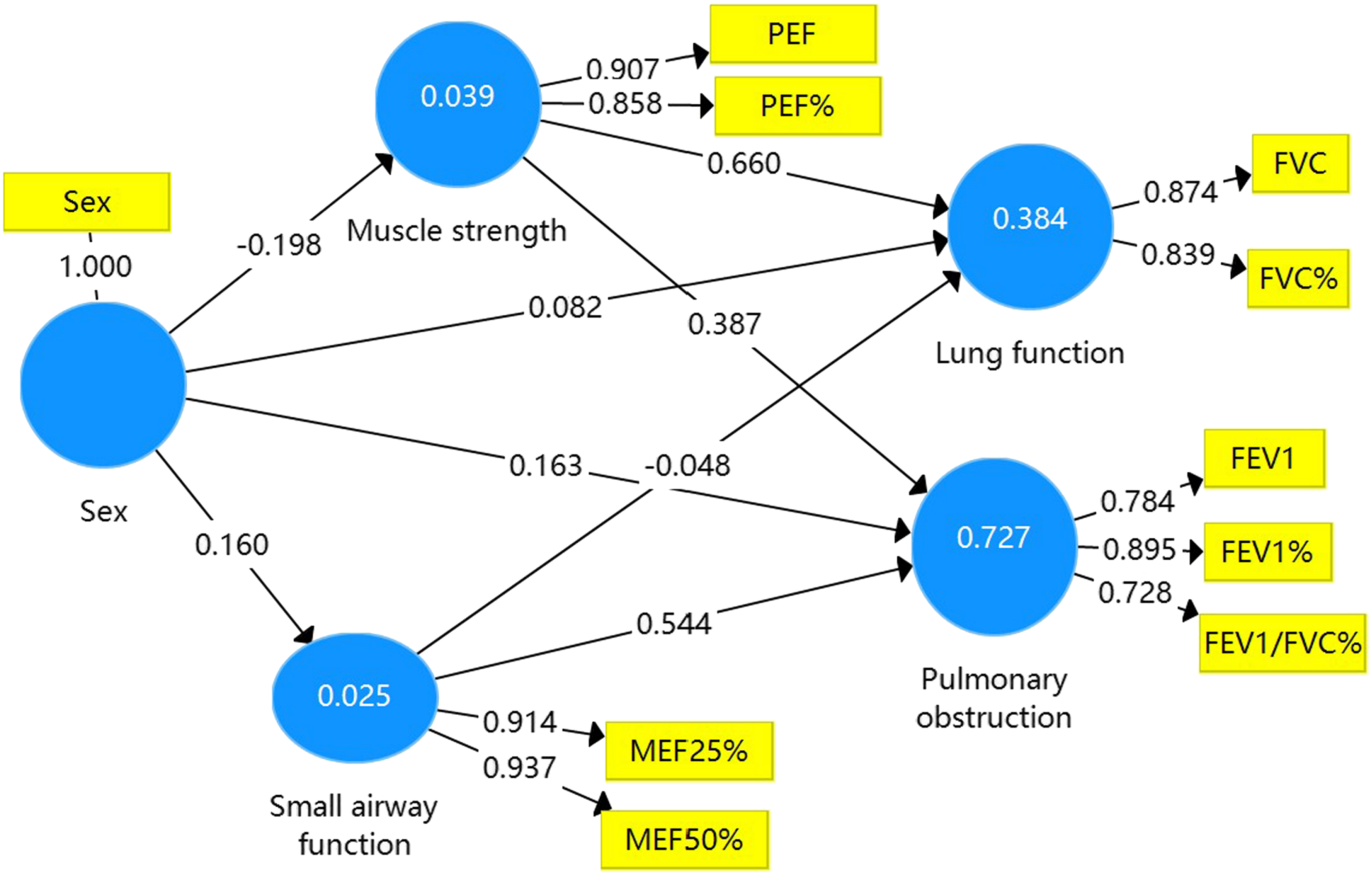
Mediating effect of sex on lung function (FVC), via muscle strength

**FIGURE 5:**
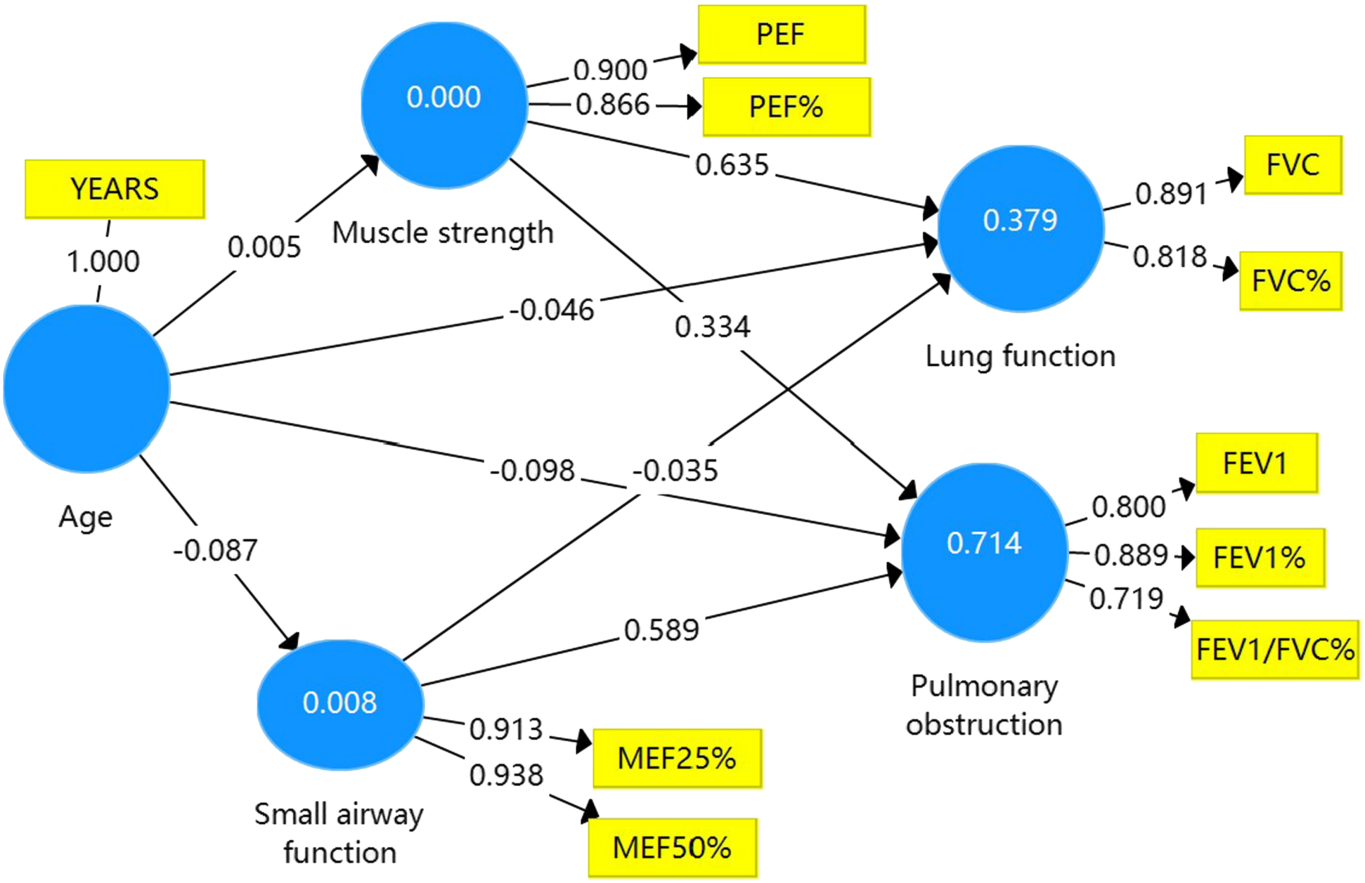
Body mass index as a moderator affects the effects of small airway function on lung function (FVC)

According to the views from Preacher and Hayes,^17^ the path model for the relationship between sex (*β* = −0.306, *p* < 0.05) and lung function was statistically significant. However, after adjusting the indirect effects of the mediator, the direct effect of sex (*β* = −0.124, *p* > 0.05) on lung function was no longer significant, as shown in Figure 4. The indirect effects indicated that there was a mediation. Figure 4 clearly depicted that sex was positively related to engagement in muscle strength, which in turn was significantly related to FVC. As a moderator, BMI increased the effects of small airway function on FVC (Figures 3 and 5).

## 4 DISCUSSION

The aim of this study was to examine the complex relationship between the confounding factors (sex, age, and BMI) and pulmonary function in patients with lung cancer utilizing the path analyses method. Our findings show that age, sex, and BMI have a small effect on the perceived lung function and pulmonary obstruction. Sex and age were positively associated with pulmonary obstruction, and only the path between sex and muscle strength was statistically significant. The results in the present study also indicated that muscle strength as a mediator between sex and lung function is novel, and BMI adjusted the effects of small airway function on FVC.

Lung structural changes occur with age.^18^ In healthy individuals without smoking, mean bronchial diameter decreases resulting in increased airway resistance with age, particularly in peripheral airways, and pulmonary function declines gradually from age 25 years.^18^ In the present study, age was positively associated with pulmonary obstruction, and small airway functions have a large effect on pulmonary obstruction. Furthermore, aging is associated with increased FVC and small airway abnormality regardless of respiratory symptoms.^3^ Those results indicated that age had a direct effect on pulmonary obstruction.

PEF is not the same to be FEV1 and FVC as a reliable lung function index and reflects the strength of abdominal muscles. We found that the relationship between sex and FVC was significantly mediated by muscle strength. Interestingly, sex differences in PEF were observed in a large sample twin study,^6^ and the results supported that sex indirectly affected lung function mediated by muscle strength.

Many studies had explored the effects of BMI on pulmonary function in different kinds of peoples. In childhood and adolescence,^5^ BMI was associated with airway obstruction including the small airway, and overweight and obesity lead to higher FVC at 8 years and lower FEV1/FVC ratio at 8 and 16 years. In patients with asthma, increased BMI predisposes to airway closure during bronchoconstriction.^8^ Interestingly, Chih et al.^19^ suggested that pulmonary function was a significant mediator of the link between central obesity and childhood asthma. However, underweight was associated with decreased pulmonary function in healthy adults and expressed lower FVC and FEV1.^9^ Those results clearly showed that the effects of BMI on pulmonary function occurred independently of the status of BMI. Unfortunately, we had no more data to explain how BMI affects small airway function and FVC. The results from our study showed that BMI as a moderator increased the effects of small airway function on FVC in patients with lung cancer.

The study has some limitations. Though the use of path analysis allows a rigorous analysis in this limited‐evidence area, we used cross‐sectional data but not longitudinal data that may have allowed us to explore causality. More experimental studies are required to confirm the finding in this study. Furthermore, we did not test the influence of these factors together on pulmonary function.

In conclusion, the structural model in preoperational patients with lung cancer revealed the different influence of contextual factors (sex, age, and BMI) on pulmonary function. Additional works are necessary to replicate these findings and to test the models under disease‐related conditions.

## ETHICS STATEMENT

This study was approved by institutional review board of the center. Informed consent was waived because this study was considered as posing minimal risk to the study participants because of its retrospective study design.

## AUTHOR CONTRIBUTIONS

XP Li analyzed and interpreted the patient data. WC Xu, YK Liu, and Bin Zeng performed the data collection. XP Li and WC Xu were the major contributors in writing the manuscript. All authors read and approved the final manuscript.

## CONFLICT OF INTEREST

The authors have no conflicts of interest to declare.

## Data Availability

The datasets used and/or analyzed during the current study are available from the corresponding author on reasonable request.
